# BRAF inhibitor monotherapy in BRAFV600E-mutated pediatric low-grade glioma: a single center’s experience

**DOI:** 10.3389/fonc.2024.1505951

**Published:** 2025-01-07

**Authors:** S. S. McThenia, K. M. Reddy, E. Damaraju, E. Castellino, Z. He, R. Beer, F. Chien, R. C. Castellino, A. E. Goldman-Yassen, J. R. Fangusaro, T. MacDonald

**Affiliations:** ^1^ Department of Pediatrics, Children’s Healthcare of Atlanta, Atlanta, GA, United States; ^2^ Department of Pediatrics, Emory University, Atlanta, GA, United States; ^3^ Aflac Cancer & Blood Disorders Center, Atlanta, GA, United States; ^4^ Department of Radiology, Children’s Healthcare of Atlanta, Atlanta, GA, United States; ^5^ Department of Radiology and Imaging Sciences, Emory University, Atlanta, GA, United States

**Keywords:** BRAFV600E-mutation, pLGG, BRAF inhibitor, monotherapy, dabrafenib, CNS tumors, outcomes

## Abstract

**Background:**

Pediatric low-grade gliomas (pLGGs) have an overall survival of over 90%; however, patients harboring a BRAF^V600E^ alteration may have worse outcomes, particularly when treated with classic chemotherapy. Combined BRAF/MEK inhibition following incomplete resection demonstrated improved outcome in BRAF^V600E^ altered pLGG compared to combined carboplatin/vincristine chemotherapy and is now considered the standard FDA-approved treatment for this group of tumors. The aim herein was to investigate the efficacy and tolerability of single agent BRAF inhibitor treatment in BRAF^V600E^ altered pLGG.

**Methods:**

A single institution retrospective chart review analysis was performed on patients, 0 to 21 years of age, with newly diagnosed and/or progressive BRAF^V600E^ mutated pLGGs (WHO Grade 1 or 2) at Children’s Healthcare of Atlanta treated off-study with BRAF inhibitor monotherapy between 2013-2023. 2-year progression free survival (PFS) and objective tumor response was evaluated. All toxicities possibly associated with BRAF inhibition therapy were evaluated and described according to Common Terminology Criteria for Adverse Events version 5 (CTCAEv5). MRI brain imaging data at baseline and best response was evaluated to identify patterns that may predict response to BRAF inhibition monotherapy.

**Results:**

Fifteen patients diagnosed with BRAF^V600E^ mutated pLGG, treated with monotherapy BRAF inhibition, were identified. Median age of diagnosis: 3.8 years (0.2 –18.1). Histologic diagnosis: pilocytic astrocytoma (PA) (N=4); ganglioglioma (GGL) (N=3); GGL, atypical (N=3); pleomorphic xanthroastrocytoma (PXA) (N=2); low-grade neuroepithelial tumor (N=1); infiltrating glioma (N=1); and LGG (NOS) (N=1). Tumor locations included: hypothalamus/optic chiasm (N=6); brainstem (N=4); third ventricle/thalamus (N=2); parietal/temporal lobe (N=2); and spinal cord (N=1). Mean duration of BRAF inhibitor monotherapy: 38.41 months (range 3.9-83.7). Median follow-up: 32.6 months (16 - 78.1). *Two-year PFS for patients on BRAFi monotherapy for at least 10 months: 90% (95% CI: 73.2%-100%). Objective Response (OR) for 15 evaluable patients on BRAF inhibitor (BRAFi) therapy: 73% (0/15 CR + 6/15 PR + 5/15 MR) with Overall Response Rate (ORR=CR+PR): 40%.* Overall, patients tolerated treatment well with Grade 1 rash being the most common toxicity. Two of 15 patients (13%) discontinued therapy due to toxicities, and 2 other patients switched within drug class from vemurafenib to dabrafenib due to toxicities.

**Discussion:**

In this small cohort of incompletely resected BRAF^V600E^ mutated pLGGs, BRAFi monotherapy was effective and well tolerated with an ORR comparable to published prospective outcomes of dual MEK/BRAF inhibitor therapy. This promising monotherapy treatment should be considered when choosing treatment for incompletely resected BRAF^V600E^-altered pLGGs.

## 1 Introduction

Pediatric low-grade gliomas (pLGG), the most common pediatric central nervous system (CNS) tumors, have an excellent outcome with a 20-year overall survival (OS) greater than 90% (1, 2). They are classified by the World Health Organization (WHO) as grade 1 and grade 2 tumors (3). Though tumorigenesis in pLGG is most commonly driven by alterations in RAS/Mitogen-activated protein kinases (MAPK) associated pathways, pLGG comprise a heterogeneous group of CNS tumors (3). Approximately 17% of pLGGs have BRAFV600E point mutations (4). BRAFV600E is a potentially highly targetable mutation (5).

The subset of pLGGs with BRAFV600E-alterations has worse outcomes than V600E-wild type (wt) tumors with less than 30% of patients with pLGG BRAFV600E alterations having tumor control with standard therapies, including chemotherapy and radiotherapy (5). Historically, standard of care of treatment for pLGG is maximal safe surgical resection followed by observation versus chemotherapy (such as carboplatin and vincristine) versus radiation therapy. Though radiation therapy results in good responses, it is associated with undesirable, long-term late effects, particularly in young children, including neurocognitive dysfunction, vascular abnormalities and secondary malignancy (2, 6, 7). Standard chemotherapy for BRAFV600E-mutated pLGG is inadequate for many patients. In a recent phase II trial comparing standard chemotherapy with carboplatin (C) plus vincristine (V) to dual MAPK/Mitogen-activated protein kinase (MEK) inhibitor therapy dabrafenib (dab) and trametinib (tram) in patients with BRAFV600E mutated pLGG treated upfront following surgical biopsy or subtotal resection, those treated with chemotherapy had a 12-month PFS of only 26%, and an Overall Response Rate (ORR) of only 11% (95% CI, 3%-25%) (4). A 2015 population-based study found that while only 2.9% of pLGGs transformed to secondary high grade gliomas (sHGG), BRAF V600E mutations were present in 44% (8 of 18) of pLLGs that transformed to sHGG as compared to 6% (10 of 167) of pLGGs that did not transform (5, 6, 8). A subset of patients with BRAFV600E altered pLGGs with a concurrent tumor suppressor gene CDKN2A co-deletion had an increased risk of malignant transformation with particularly poor outcomes (2, 5, 8–11).

With the goal of improving outcomes in patients with BRAFV600E mutated pLGGs, oral adjuvant therapy is used to target alterations in the MAPK (mitogen-activated protein kinases) pathway, specifically, by using a MEK inhibitor (MEKi), in combination with first generation type 1 BRAF inhibitor (BRAFi) (4). Data from phase I/II studies with dual BRAF/MEK inhibitor (dab + tram) therapy not only demonstrated early signs of efficacy but also safety (4, 12). In a primary analysis of a phase II trial of dab + tram in BRAFV600E-mutant pLGGs, the dual inhibitor therapy significantly increased ORR using RANO criteria, prolonging PFS compared to standard of care chemotherapy (C + V), noting an ORR of 47% (95% CI, 35%-59%) with dab + tram vs 11% (95% CI, 3%-25%) with C + V (P<0.001), odds ratio, 7.2 (95% CI, 2.3-22.4), and 12-month PFS 67% with dab + tram vs 26% with C+ V (4, 12). In addition to improved efficacy, those who received the dual inhibitor therapy (dab + tram) had fewer grade > 3 adverse events (AEs; 47%) as compared to those in who received standard of care chemotherapy (C + V) (AEs; 18%) (4). In March 2023, the FDA approved dual BRAF/MEK inhibitor (dab + tram) therapy in children with BRAFV600E pLGG (13).

Prior to the FDA approval of BRAF/MEK combination therapy, Children’s Healthcare of Atlanta had used monotherapy BRAFi off-study in patients with many BRAFV600E mutated pLGGs. Several early phase clinical trial suggested efficacy and tolerability in BRAFi monotherapy. A phase I/II multicenter study of advanced BRAF V600E mutated solid tumors (NCT01677741) included 32 patients with BRAF V600E pLGGs treated with dabrafenib monotherapy, finding meaningful clinical activity and acceptable tolerability (14). A phase I study through the Pacific Pediatric Neuro-Oncology Consortium study (PNOC-002) treated children with recurrent or progressive BRAFV600E –mutated brain tumors with monotherapy BRAFi with vemurafenib, showing promising anti-tumor activity with manageable toxicity (15). Yet, despite these two early phase studies, limited additional published data exists to further describe the outcomes of monotherapy with BRAFi for BRAFV600E mutated pLGGs. Herein reports data from a single institution retrospective chart review of patients with BRAFV600E mutated pLGGs, who were treated off-study with BRAFi monotherapy. This review included both patients started on BRAFi therapy at diagnosis following diagnostic biopsy or sub-total resection, as well as those treated for subsequent progressive disease. The pathological features, imaging responses, treatment-related toxicities, and clinical outcomes were collected and analyzed.

## 2 Materials and methods

### 2.1 Methods

Clinical data was obtained, following IRB approval, from the electronic medical records of Children’s Healthcare of Atlanta (CHOA), which longitudinally documents all patients diagnosed with pLGG and treated at CHOA between 2013 and 2023. pLGG was defined as any glial or mixed glial neuronal tumor, excluding ependymoma, graded as a Grade 1 or 2 by the WHO Classification of Tumors of the Central Nervous System (CNS), 5th Edition. In addition, patients needed (1) a diagnosis of having a pLGG with a BRAF^V600E^ alteration confirmed by either immunohistochemistry or sequencing, (2) received treatment with BRAFi monotherapy, and (3) had measurable disease at the start of BRAFi monotherapy. RAPNO criteria was used (16). Measurable disease was defined as visible in three standard planes, with a diameter of at least 10 mm in each plane on T2-weighted and T2-weighted fluid-attenuated inversion recovery imaging (FLAIR) (16).

Dabrafenib PO 5.25Mg/kg/day. Dose divided BID. (< 12 year old); 4.5 mg/kg/day. Dose divided BID. (=>12 year old). Maximum dose: 300 mg/day divided BID.

Pediatric patients diagnosed at age 21 years or younger with a BRAF^V600E^ mutated pLGG were included for retrospective analysis. The diagnosis, location of tumor, extent of surgical resection, number and description of prior lines of chemotherapy and/or radiation therapy, outcomes, and toxicities associated with BRAFi, specifically vemurafenib and/or dabrafenib, monotherapy treatment were assessed. Starting dose of vemurafenib PO was 550mg/m2/dose twice a day (maximum dose 960 mg twice a day), and starting dose of dabrafenib PO was 5.25mg/kg/day dose divided twice a day (<12 years of age); 4.5 mg/kg/day dose divided twice a day (=>12 years of age) (maximum dose 300 mg/day). To assess response to BRAFi monotherapy, MRI brain imaging findings were compared from diagnosis and/or prior to start of BRAFi therapy, at 3 months, 6 months, and 1 year after the initiation of treatment, as well as MRI at best response if greater than 1-year in BRAFi therapy.

In order to evaluate for patterns that may predict positive response to BRAFi monotherapy, analysis of MRI findings at the start of therapy and at the best response was collected. On each MRI brain scan, experienced pediatric neuroradiologists collected: signal intensity on pre-contrast T1-weighted and T2-weighted images, apparent diffusion coefficient (ADC) mean and standard deviation in the solid portion of the tumor, enhancement pattern, presence of cystic or solid components, presence of peritumoral edema, and presence of hemorrhage. Intensity features were categorized as hypo-intense, iso-intense, or hyperintense relative to white matter. Enhancement was categorized as absent, heterogeneous, or avid. Cystic and solid components were categorized as either solid or cystic and solid. Peritumoral edema and hemorrhage were categorized as either present or absent.

### 2.2 Statistical analysis

Descriptive analysis was summarized. PFS was defined as the interval between start of BRAFi monotherapy and time of radiologic or clinical progression. PFS was analyzed by the Kaplan-Meier method and p values are reported using the log-rank test. Survival data are presented as survival estimates, including 95% confidence intervals (CI).

Adverse events were noted based on documented in the electronic medical record, as well as noting when necessitating dose reduction, and/or discontinuation of BRAFi therapy. Previous treatment with chemotherapy regimens and prior treatment with radiation was assessed.

Response was evaluated according to reduction in tumor size, as measured by the product of two dimensions on T2 or T2 fluid-attenuated inversion recovery (FLAIR) magnetic resonance imaging (MRI) for low-grade gliomas Response Assessment in Pediatric Neuro-Oncology criteria for pLGG (16–18). Minor response (MR) was defined as reduction in tumor size between 25% and 49%, partial response (PR) as reduction in tumor size between 50% and 99%, and complete response (CR) as disappearance of disease on FLAIR or T2 imaging. Progressive disease (PD) was defined as either any tumor growth or clinical deterioration, as judged by treating physician, requiring change in management. Objective response (OR) was defined as reduction in tumor size of ≥ 25%, and overall response rate (ORR) was determined by combining PR and CR (19, 20). Median time to best response was evaluated at available MRIs during BRAFi monotherapy treatment. Imaging data was analyzed to determine what biomarkers predict positive response to therapy. Frequencies were calculated for categorical imaging variables, and averages and standard deviation were calculated for ADC values.

For the MRI imaging analysis, univariate statistics comparing imaging variables between the three best response categories were performed using Stata version 18.0 (College Station, TX). Categorical variables were compared using the Fisher’s exact or Chi-squared test, when appropriate. Continuous variables were assessed for normality by visual inspection of histograms or the Shapiro-Wilk Test. Nonparametric Kruskal–Wallis test or parametric ANOVA tests were used when appropriate. Two-tailed p-values less than 0.05 were considered statistically significant.

## 3 Results

### 3.1 Patient characteristics

Overall, the cohort included 15 evaluable patients with BRAF^V600E^mutated pLGG treated off-study with BRAFi monotherapy at CHOA. Demographics and clinical characteristics of the 15 patients included in this retrospective review are summarized in **Table 1**. Of the 15 patients, the median age of BRAF^V600E^ mutated pLGG diagnosis was 3.8 years, range (0.2-18.1 years). The most common tumor location among the 15 patients was hypothalamus/optic chiasm (6/15), followed by brainstem (4/15), 3^rd^ ventricle/thalamus (2/15), temporal/parietal lobe (2/15) and spinal cord (1/15). Tumor histopathology at diagnosis demonstrated the heterogeneity of BRAF^V600E^ pLGGs with pilocytic astrocytoma (PA) (N=4); ganglioglioma (GGL) (N=3); GGL, atypical (N=3); pleomorphic xanthroastrocytoma (PXA) (N=2); low grade neuroepithelial tumor (N=1); infiltrative glioma (N=1); low grade glioma (NOS) (N=1). One third of the patients had a sub-total resection and 2/3 of the patients underwent biopsy only. At the start of BRAFi monotherapy, all patients had “measurable disease”, defined by RAPNO criteria (16, 20).

**Table 1:** Clinical characteristics of pediatric low-grade glioma patients receiving BRAF inhibition (BRAFi) monotherapy at Children’s Healthcare of Atlanta.

Characteristic	N*
Total	15
Sex	
Female	8
Male	7
Age at diagnosis, years	
Median	3.8
Range	0.2-18.1
Surgery type	
Resection, subtotal	5
Biopsy only	10
Tumor location	
Hypothalamus/Optic chiasm	6
3^rd^ ventricle/Thalamus	2
Brainstem	4
Spinal cord	1
Parietal/Temporal lobe	2
Histopathology diagnosis	
Pilocytic astrocytoma (PA	4
Ganglioglioma (GGL)	3
GGL, atypical	3
Pleomorphic xanthroastrocytoma (PXA)	2
Low grade neuroepithelial tumor	
Infiltrative glioma	1
Low grade glioma (NOS)	1
Prior lines of chemotherapy	1
0	10
1	2
2-4	3
Radiation prior to BRAFi monotherapy	
No	14
Yes	1
Reason for starting BRAFi monotherapy	
Neurologic signs/symptoms following Initial diagnosis	8
Progression of disease on or after prior treatment **	5
Progression of disease after observation	2

*Unless noted otherwise.

**See **Table 2** for further details.

#### 3.1.1 Prior treatments

Of the 15 patients treated with BRAFi monotherapy, 6 patients were previously treated, 5 with chemotherapy and 1 with radiotherapy. See **Table 2** for description of prior lines of chemotherapy. Two of the 5 patients had 1 line of chemotherapy prior to starting BRAFi treatment, and 3 of the 5 patients had 2-4 prior lines of chemotherapy. 5 of the 6 patients who received prior chemotherapy or radiotherapy were started on BRAFi therapy due to progression of disease on MRI imaging. The other patient, an infant, was temporarily started on chemotherapy while awaiting approval for BRAFi due to persistent neurological signs and symptoms and ongoing risk of neurological decline.

**Table 2:** Description of lines and names of chemotherapy patients received prior to BRAFi therapy initiation.

Patient #	Chemotherapy line 1	Reason for stopping line 1	Line 2	Reason for stopping line 2	Line 3	Reason for stopping line 3	Line 4	Reason for stopping line 4
1	Carboplatin and vincristine per CCG A9952 Regimen A	Allergic reaction to carboplatin 3.5 mo on treatment	Vinblastine, weekly	PD 5.5 mo on treatment				
9	Carboplatin and vincristine per CCG A9952 Regimen A	Toxicity to carboplatin with PD 4 mo after stopping treatment						
10	Cyclophosphamide and vincristine per Baby POG, Cycle A	Approved for BRAFi after 1 Cycle of A						
14	Carboplatin and vincristine per CCG A9952 Regimen A	PD 2 mo after completion of treatment	Phase II trial of Poly- ICLC	PD 6 mo after completion of treatment	Avastin and irinotecan NOS	Persistent hypertension 7 months on treatment		
15	Carboplatin per CCG A9952 Regimen A (without vincristine due to patient’s age < 1 year old)	PD 13 mo on therapy	TPCV per CCG A9952 Regimen B	SD for 12 mo on treatment and PD 14 mo after completion of therapy	Vinblastine, weekly	PD 4.5 mo on treatment	Phase II trial of Poly-ICLC, enrolled	PD 11 mo on treatment

CCG, Children’s Cancer Group; PD, progressive disease; SD, stable disease; TPCV, chemotherapy regimen combines thioguanine (T), procarbazine (P), CCNU (C), lomustine, and vincristine (V); NOS, Not on study; poly-ICLC, synthetic immunostimulant that is a combination of polyinosinic-polycytidylic acid (poly I:C), carboxymethylcellulose, and poly-L-lysine.

Nine of the 15 patients treated with BRAFi monotherapy had no prior chemotherapy or radiation therapy. Of these 9 patients, 7 started BRAFi treatment following diagnostic biopsy due to persistent neurological signs and symptoms and ongoing risk of neurological decline. The other 2 of 9 patients were initially observed following diagnostic biopsy and were started on BRAFi treatment following subsequent progressive disease noted both on MRI imaging and clinical exam.

### 3.2 BRAFi toxicities

As patients treated with BRAFi monotherapy were not enrolled in a clinical trial, toxicities described in the electronic medical record on chart review that may have been associated with BRAFi therapy were noted and evaluated by CTCAEv5 criteria. During BRAFi monotherapy, 9 patients experienced drug-related toxicities of any grade (1-5). Toxicities and treatment details are summarized in **Table 3**. Most toxicities were Grade 1 or 2, yet all 9/15 patients (60%) who experienced drug toxicities had at least 1 dose reduction and/or temporary hold of their BRAFi therapy because of adverse effects. The most common toxicity was rash with 6 patients experiencing a Grade 1 rash and 3 patients having a Grade 2 rash. Other Grade 1 toxicities included hair loss (n=3), headache (n=1), diarrhea (n=1), and fever (n=1). Additional Grade 2 toxicities included arthralgia (n=1), myalgia (n=1), photosensitivity (n=1), fatigue (n=1), headache (n=1), xerosis (n=1), and corneal lesion (n=1). One patient experienced Grade 3 toxicity with elevated liver function tests (LFTs). Two patients permanently discontinued BRAFi monotherapy because of toxicities.

**Table 3:** Toxicities and treatment details of BRAF^V600E^ mutated pLGG receiving BRAFi monotherapy at Children’s Healthcare of Atlanta.

Patient	Treatment round	Targeted therapy	Timing of BRAFi start from initial diagnosis, months	Toxicity (CTCAE grade)	Dose reduction for toxicity (reason)	Treatment cessation (reason)	Time on therapy, months
**1**	1	Vemurafenib	16.0	Photosensitivity (2) Rash (2)	Yes (Rash)	Yes (Toxicity)	53.0
	2	Dabrafenib	–	No	No	No	19.3
**2**	1	Dabrafenib	27.1	No	No	Yes (End of treatment)	39.0
	2	Dabrafenib	–	No	No	No	6.7
**3**	1	Dabrafenib	1.7	Rash (1)	Yes (Rash)	No	14.1
**4**	1	Dabrafenib	4.0	No	No	No	39.9
**5**	1	Dabrafenib	1.3	No	No	Yes (PD)	9.6
**6**	1	Dabrafenib	2.0	Rash (1)	Yes (Rash)	No	63.4
**7**	1	Dabrafenib	0.5	Rash (1) Hair loss (1) Fatigue (2) Headache (2)	Yes (Rash, fatigue, headache)	No	27.7
**8**	1	Dabrafenib	25.3	Rash (1)	Yes (Skin)	No	11.2
**9**	1	Dabrafenib	28.0	No	No	No	45.7
**10**	1	Dabrafenib	1.7	No	No	No	67.8
**11**	1	Vemurafenib	1.3	Rash (1) Headache (1) Hair loss (1)	Yes (Skin)	Yes (Toxicity)	5.8
	2	Dabrafenib	–	Corneal lesion (2) Xerosis (2)	Yes (Eye)	Yes (Toxicity)	2.8
**12**	1	Dabrafenib	1.5	Rash (1) Elevated LFTs (3)	Yes (Skin, increased LFTs)	No	83.7
**13**	1	Vemurafenib	14.1	No	No	Yes (End of treatment)	12.7
	2	Vemurafenib	–	Hair loss (1) Diarrhea (1) Rash (2) Arthralgia (2)	Yes (Rash, arthralgia, diarrhea)	Yes (End of treatment and toxicity)	14.5
	3	Vemurafenib	–	No	No	Yes (Toxicity)	6.4
	4	Vemurafenib	–	No	No	No	41.5
**14**	1	Dabrafenib	70.5	Fever (1) Rash (2) Arthralgia (2) Myalgia (2)	Yes (Rash, arthralgia, myalgia)	Yes (Toxicity)	3.9
**15**	1	Vemurafenib	62.3	No	No	Yes (PD)	6.2
	2	Dabrafenib	–	No	No	Yes (PD)	1.2

PD, progression of disease.

Treatment round: the time from the of specific BRAFi monotherapy to the last day of that specific BRAFi.

### 3.3 Patient outcomes

The Objective Response (OR=CR+PR+MR) rates for 15 evaluable patients on BRAFi therapy in our study was 73% (0/15 CR + 6/15 PR + 5/15 MR) and Overall Response Rate (ORR= CR+PR) was 40% (0/15 CR + 6/15 PR). Average time to best response (MR, PR, or CR) was 11.13 months (1.7 – 61.3) with a median time to best response of 6.3 months. See **Table 4** for details of best response. 40% (6/15) patients had achieved best response by 6 months of therapy and 67% (10/15) patients had achieved a best response by 1 year of therapy. Most patients with pLGGs had sustained response with a median treatment time of 38.41 months (range 3.9-83.7). Median follow-up: 49.5 months (12.4 – 96.3).

**Table 4:** Best response of pediatric low-grade glioma patients (based on RAPNO criteria) receiving BRAF inhibition monotherapy at Children’s Healthcare of Atlanta.

Patient	Best response	Time, months*
1	Partial	61.3
2	Partial	14.0
3	Minor	2.5
4	Minor	1.7
5	Partial	6.5
6	Partial	6.3
7	Partial	9.0
8	Stable	3.5
9	Minor	6.3
10	Stable	13.0
11	Stable	5.5
12	Minor	13.3
13	Partial	5.9
14	Minor	15.3
15	Stable	2.9

*Time from BRAFi monotherapy initiation to best response.

For the 4 patients with best response of stable disease (SD) in **Table 4**, patient 8 continues to have SD at 11.2 months on BRAFi monotherapy; patient 10 continues to have SD at 67.8 months *on* BRAFi monotherapy; patient 11 continued with SD for 28 months *following* stopping 5.5 mo of BRAFi monotherapy due to toxicities before having PD off treatment; and patient 15 had SD at 6.2 months *on* BRAFi monotherapy prior to having PD *on* treatment.

**Figure 1:**
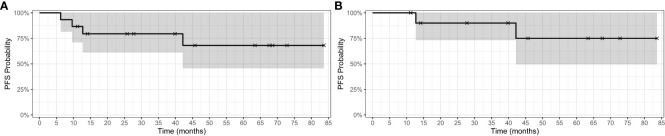
Kaplan-Meier survival curve for BRAF^V600E^-mutated pLGGs. **(A)** Kaplan-Meier PFS curve for 15 patients with BRAF^V600E^ mutated pLGGs treated with BRAF monotherapy inhibition **(B)** Kaplan-Meier PFS curve for the 11 patients treated ≥ 10 months with BRAF^V600E^ mutated pLGGs treated with BRAFi monotherapy.

**Figure 2:**
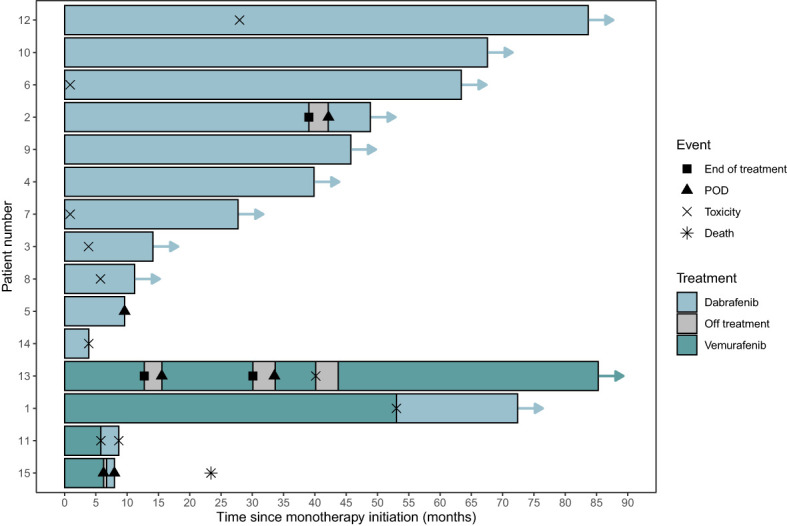
Swimmer plot for BRAF^V600E^ mutated pLGGs treated with monotherapy BRAFi. Legend describes parameters of response, progression, and ongoing therapy. CR, complete response; MR, minor response; POD (PD), progressive disease; PR, partial response; SD, stable disease.

The estimated one- and two-year PFS for the 15 patients was 86.7% (95% CI 71.1% - 100%) and 79.4% (95% CI 61.2%-100%), respectively. (**Figure 1A**) The estimated one- and two-year PFS for the 11 patients treated on BRAFi monotherapy for *at least 10 months* was 95% (95% CI 100%-100%) and 90% (95% CI 73.2%-100%), respectively. (**Figure 1B**) Eleven of the 15 patients (73%) currently remain on BRAFi monotherapy. Progression of disease occurred in 2 of 15 patients while on BRAFi monotherapy treatment, one of whom was switched to a different BRAFi but quickly progressed without evidence of a response (See details in **Figure 2**). **Figure 2** uses a swimmer plot to describe the individual trajectories of each of the 15 patients treated with BRAF^V600E^ mutated pLGGs treated with monotherapy BRAFi describing parameters of response, progression, and ongoing therapy.

Results of comparison of baseline MRI brain imaging results prior to initiation of BRAFi monotherapy (**Table 5**) to subsequent MRI brain imaging results at time of best response on BRAFi monotherapy (**Table 6**) are summarized in **Table 7**. **Table 7** describes the proportions of tumor imaging findings that changed between the baseline and best response. Comparison between baseline and best response demonstrates that T2-weighted signal was more likely to be hyperintense on baseline scans of subjects who demonstrated minor or partial response compared with stable tumor (p=0.009). Other imaging findings were not significantly different between response groups on both initial scans and on the scan of best response. This was still true when minor and partial response were grouped. No significant difference in tumor response was found based on how the tumor imaging findings changed between the baseline and scan of best response.

**Table 5:** Baseline MRI brain tumor results, prior to initiation of BRAFi monotherapy.

	Stable Response (n=4)	Minor Response (n=5)	Partial Response (n=6)	p-value (test)
Volume; median (product of 3 plane measurements in cm) (IQR)	4 (1-14)	33 (7-51)	48 (16-364)	0.095*
T1-weighted signal; percent (n)				0.348**
Hypointense	50% (2)	60% (3)	50% (3)	
Iso-hypointense	50% (2)	40% (2)	33% (2)	
Isointense	0% (0)	0% (0)	17% (1)	
T2-weighted; percent (n)				0.009**
Hyperintense	25% (1)	100% (5)	100% (6)	
Iso-hyperintense	25% (1)	0% (0)	0% (0)	
Isointense	50% (2)	0% (0)	0% (0)	
Enhancement; percent (n)				0.604**
Heterogeneous	50% (2)	80% (4)	83% (5)	
Avid	50% (2)	20% (1)	17% (2)	
ADC mean; median (IQR)	855 (794-1032)	1129 (1015-1316)	1090 (1018-1176)	0.232*
ADC SD; median, (IQR)	64 (38-73)	65 (49-78)	118 (60-141)	0.329*
Tumor texture; percent (n)				0.650**
Solid	25% (1)	20% (1)	50% (3)	
Cystic and solid	75% (3)	80% (4)	50% (3)	
Surrounding T2-weighted hyperintense signal; percent (n)	50% (2)	80% (4)	50% (3)	0.660**
Hemorrhage; percent (n)	75% (3)	60% (3)	50% (3)	0.820**

*Kruskal–Wallis; **Fisher exact test Apparent diffusion coefficient (ADC); Standard deviation (SD); Interquartile range (IQR).

**Table 6:** Best response MRI brain tumor results, while treated with BRAFi monotherapy.

	Stable Response (n=4)	Minor Response (n=5)	Partial Response (n=6)	p-value (test)
T1-weighted signal; percent (n)				0.960**
Hypointense	25% (1)	20% (1)	0% (0)	
Iso-hypointense	0% (0)	20% (1)	33% (2)	
Isointense	50% (2)	40% (2)	50% (3)	
Iso-hyperintense	25% (1)	20% (1)	17% (1)	
T2-weighted signal; percent (n)				0.944**
Hyperintense	25% (1)	60% (3)	67% (4)	
Iso-hyperintense	25% (1)	20% (1)	17%(1)	
Isointense	25% (1)	20% (1)	17% (1)	
Iso-hypointense	25% (1)	0% (0)	0% (0)	
Enhancement; percent (n)				0.940**
Minimal	25% (1)	20% (1)	50% (3)	
Heterogeneous	50% (2)	60% (3)	33% (2)	
Avid	25% (1)	20% (1)	16% (1)	
ADC mean; (median, IQR)	977 (921-1114)	1287 (1190-1305)	971 (901-1060)	0.245*
ADC SD; (median, IQR)	76 (62-123)	88 (67-130)	91 (71-94)	0.695*
Tumor texture; percent (n)				0.349**
Solid	25% (1)	80% (4)	67% (4)	
Cystic and solid	75% (3)	20% (1)	33% (2)	
Surrounding T2-weighted hyperintense signal; percent (n)	100% (4)	80% (4)	67% (4)	0.736**
Hemorrhage; percent (n)	75% (3)	80% (4)	80% (4)	0.999**

*Kruskal–Wallis; **Fisher exact test.

ADC, Apparent diffusion coefficient; SD, Standard deviation; IQR, Interquartile range.

**Table 7:** Change in imaging characteristics between baseline and best response MRI brain imaging scans.

T1 increase percentage (n)	87% (13)
T2 decrease percentage (n)	53% (8)
Decreased enhancement percentage (n)	40% (6)
Increased enhancement percentage (n)	20% (3)
Cystic/solid to solid percentage (n)	40% (6)
Increased hemorrhage percentage (n)	40% (6)

**Figure 3:**
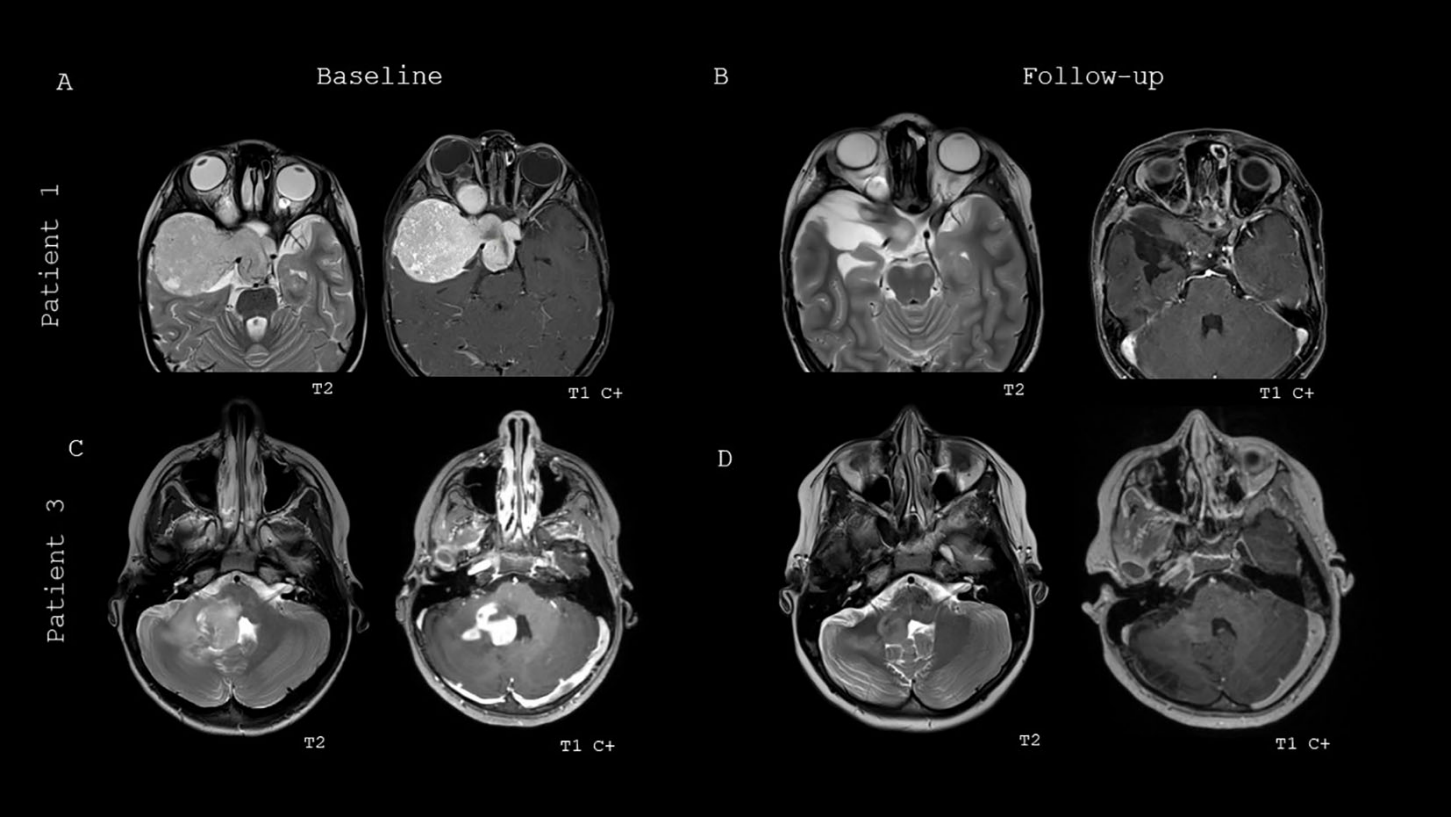
Baseline and best response MRI brain imaging of two patients treated with BRAFi monotherapy. **(A)** Baseline MRI brain imaging of patient 1 prior to start of BRAFi therapy. **(B)** Best response imaging of patient 1 on BRAFi therapy. **(C)** Baseline MRI brain imaging of patient 3 prior to start of BRAFi therapy. **(D)** Best response imaging of patient 3 on BRAFi therapy.

**Figure 3** details two patient’s response to therapy as shown with MRI brain at baseline prior to initiation of BRAFi monotherapy and follow-up images at the time of best response to BRAFi therapy. Patient 1 has an optic pathway pLGG that was partially resected at time of diagnosis, and subsequently started on BRAFi monotherapy when noted to have PD on this brain MRI while *on* his 2^nd^ line of chemotherapy (**Figure 3A**). Follow-up imaging at time of best response on BRAFi monotherapy showed overall decreased size, T2 signal and enhancement, resulting in a partial response (PR) (**Figure 3B**). Patient 3 has a brainstem and cerebellar pLGG that was biopsied at diagnosis and started on BRAFi monotherapy due to persistent neurological symptoms and risk of progressive neurological decline (**Figure 3C**). Patient 3 demonstrated a best response of minor response (MR), with overall decreased T2 signal and enhancement (**Figure 3D**).

## 4 Discussion

This report describes single institution outcomes and drug-associated toxicities of BRAF^V600E^ mutated pLGGs treated with BRAF monotherapy inhibition. Previously published outcome and toxicity data for patients treated with BRAFi monotherapy is limited. In this single institution cohort of 15 patients, documented objective tumor response (MR or greater) to monotherapy BRAFi was identified in the majority of the patients. BRAFi targeted monotherapy had an ORR (CR + PR) of 40%, which is comparable to published results of dual inhibitor therapy (dab + tram) with an ORR of 47% in 73 patients, and superior to chemotherapy (C + V) that has demonstrated an ORR of 11% in 37 patients (4, 21). These retrospective, single institutional results of BRAFi monotherapy demonstrated a median one- and two-year PFS of 86.7% and 79.4%, respectively, which compares favorably to the published 1-year results of 67% (dab + tram) vs 26% with chemotherapy (C + V) (4).

Results of analysis of MRI brain, both at start of BRAFi monotherapy and at time of best response, demonstrated that T2-weighted signal was more likely to be hyperintense on baseline scans of subjects who demonstrated minor or partial response compared with stable tumor (p=0.009). Other than T2-weighted signal, no other imaging findings at time of start of BRAFi therapy and time of best response, including T1 pre-contrast intensity, ADC mean acquired from solid tumor, enhancement pattern, presence of cystic or solid components, presence of peritumoral edema, and presence of hemorrhage, identified any patterns to predict positive response to BRAFi monotherapy. Future radiomic studies could potentially add to patient selection and response evaluation.

Notably and unexpectedly, in this single institution, off-study experience of monotherapy with BRAFi therapy, only 7% (1/15) of patients were noted to have a Grade 3 or higher adverse event, while in combination therapy (Dab + Tram), 47% of patients were reported to experience a Grade 3 or higher adverse event vs 94% with chemotherapy, suggesting that BRAFi may be a better choice than dual inhibitor therapy due to lower toxicities (4, 21).

Though BRAFi monotherapy may be associated with hyperproliferative cutaneous events, including squamous cell carcinoma (SCC) and keratoacanthoma, through BRAFi-induced paradoxical activation of MAPK pathway signaling in BRAF wild-type cells, none of the 15 patients in this review were diagnosed with SCC or keratoacanthoma during the time of follow-up (22). In this review, the most common toxicity was rash with 60% (9/15) of patients having a Grade 1 or 2 rash. In contrast, in the published results of the randomized Phase II study (NCT02684058) of first-line dab + tram vs C+V in BRAF V600–mutant pLGG, the most common toxicity in the 73 patients on the dab + tram arm was pyrexia (68%), followed by headache (47%), and vomiting (34%) (4, 18). Other Grade 1 toxicities in our retrospective review included hair loss (n=3; 6.7%), headache (n=1; 6.7%), diarrhea (n=1; 6.7%), and fever (n=1; 6.7%). Additional Grade 2 toxicities included arthralgia (n=1; 6.7%), myalgia (n=1; 6.7%), photosensitivity (n=1; 6.7%), fatigue (n=1; 6.7%), headache (n=1; 6.7%), xerosis (n=1; 6.7%), and corneal lesion (n=1; 6.7%). One patient experienced a Grade 3 toxicity with elevated liver function tests (LFTs). Two patients permanently discontinued BRAFi monotherapy because of toxicities.

Limitations of this review included a small sample size of patients. This single institutional review at CHOA was limited to 15 patients, whereas the randomized Phase II study (NCT02684058) of first-line dab + tram vs C+V in BRAF V600–mutant pLGG had 73 patients on the dab + tram arm and 37 patients on the C + V arm (4, 21). In addition, in this review, the patients were not enrolled in a clinical trial and thus the data was collected in a retrospective manner. Formal Adverse Event reporting was not formally documented, but collected through a thorough review of EMR. As a result, toxicity data in this review may appear incomplete. Additionally, while radiologic response to BRAFi monotherapy was evaluated, measurable clinical response, such as changes in eye exams or neurologic exams, was not readily available. Although clinical data was not documented in a consistent and objective manner, the progress notes indicate that patients who continued on BRAFi monotherapy were clinically stable or improved as assessed by the provider and documented physical exams. In addition, though all patients in this review had a BRAF^V600E^ mutated pLGG confirmed by either immunohistochemistry or sequencing, there was no central review of the diagnosis or imaging, and additional molecular data was not reported.

In summary, the data presented in this single institution retrospective review for pediatric patients with incompletely resected BRAF^V600E^-mutated LGG treated with BRAFi monotherapy demonstrated that in this small number of 15 patients, molecularly targeted monotherapy with BRAF^V600E^ inhibition is efficacious and overall well tolerated. Compared to the current FDA-approved dual BRAF/MEK inhibitor regimen for this patient population, results herein suggest BRAFi monotherapy could be a less toxic therapy with equivalent efficacy with potential reduced drug costs. Still to be investigated in larger studies is ideal length of treatment, risk of rebound growth once off of therapy, and risk of resistance developing with monotherapy, though potentially less risk in low grade gliomas as compared to high grade gliomas. BRAFi monotherapy for BRAF^V600E^-mutated LGG shows promising results and should be considered when choosing treatment for incompletely resected BRAF^V600E^-altered pLGGs.

## Data Availability

The original contributions presented in the study are included in the article/supplementary material. Further inquiries can be directed to the corresponding author/s.
